# Vitamin E concentration in breast milk in different periods of lactation: Meta-analysis

**DOI:** 10.3389/fnut.2022.1050011

**Published:** 2022-11-10

**Authors:** Yuandi Xi, Xianyun Wang, Kuo Liu, Huanmei Zhang, Xiangnan Ren, Ai Zhao, Yuexin Yang, Jianqiang Lai, Rong Xiao

**Affiliations:** ^1^Beijing Key Laboratory of Environmental Toxicology, School of Public Health, Capital Medical University, Beijing, China; ^2^China-DRIs Research Group on Human Milk Composition, Beijing, China; ^3^National Institute for Nutrition and Health, Chinese Center for Disease Control, Beijing, China; ^4^Key Laboratory of Human Milk Science, Chinese Center for Disease Control and Prevention, Beijing, China; ^5^Wanke School of Public Health, Tsinghua University, Beijing, China

**Keywords:** vitamin E, alpha-tocopherol, breast milk, lactation, meta-analysis

## Abstract

**Objective:**

This study systematized information about vitamin E concentration in healthy breast milk during different stages of lactation in order to support the strategies of protecting postpartum women and infants.

**Methods:**

Studies published before April 30th, 2021, which detected vitamin E concentration in breast milk of healthy women by High Performance Liquid Chromatography (HPLC) or Ultra High Performance Liquid Chromatographic (UHPLC), were evaluated. The databases of CNKI (Chinese), WanFang Data (Chinese), VIP (Chinese), PubMed, Cochrane Library, Web of Science and Embase were searched. The random effect models were used to conduct meta-analysis by the statistical software package Stata 14.0.

**Results:**

In all 4,791 searched publications, 53 with full text were selected, which included 46 descriptive studies, 1 case-control study, 1 non-randomized controlled trial, and 5 randomized controlled trials. The pooled mean of vitamin E concentration was 10.57 mg α-TE/L (95%CI 8.94–12.20) in colostrum, 4.03 mg α-TE/L (95%CI 3.29–4.77) in transitional milk and 3.29 mg α-TE/L (95%CI 2.95–3.64) in mature milk. Subgroup analysis showed that vitamin E concentration of colostrum in Asian countries was lower than that in Western countries in colostrum and transitional milk.

**Conclusions:**

Vitamin E concentration in breast milk decreased during lactation until the mature milk was produced. The vitamin E concentration of colostrum in Asian countries was evidently lower than that in Western countries. The vitamin E concentration in mature milk is similar in different regions. The concentration of vitamin E in breast milk started to be stable from about 2 to 3 weeks postpartum until 4 or 6 months postpartum, but it needs additional evidence to support.

## Introduction

Breast milk is important for infant growth and development, which is the most convenient and accessible source of nutrition for infants in the first 6 months of life. The exclusive breastfeeding is recommended for the first 6 months and then continued breastfeeding alongside appropriate complementary foods from thereafter to 24 months. Consequently, studying the composition of breast milk is of crucial importance (1, 2).

Vitamin E, also known as tocopherol, functions as a potent antioxidant, which protects cells from oxidative damage and maintains normal immunity. It is closely related to the development of respiratory, immune and cognitive systems in infants (3). It comprises a group of compounds possessing tocopherol and tocotrienol and their derivatives. Vitamin E includes four tocopherols and four tocotrienols designated as α-, β-, γ-, and δ-. α-tocopherol, which is preferentially recognized by the α-tocopherol transfer protein (TTP) in the human body, is the compound playing the highest vitamin E activity (4, 5).

The nutritional supplement of vitamin E to the fetus through the placenta is limited during pregnancy. Therefore, postpartum breastfeeding has become a significant source for infants to obtain vitamin E. This way of vitamin E supplementation could help infants defend oxygen toxicity in the extrauterine environment and protect the lipoproteins and polyunsaturated fatty acids present in the cellular membranes against peroxidation (6). As described above, the content of vitamin E in breast milk is extremely vital for babies.

This review systematically searched and analyzed three databases for Chinese language articles, four databases for English language articles to obtain more comprehensive information. The aim of the present meta-analysis was to systematize information about vitamin E concentration in healthy breast milk during different periods of lactation, which might be useful to establish support strategies to protect postpartum women and infants.

## Materials and methods

This meta-analysis was conducted according to the norms of Meta-Analysis of Observational Studies in Epidemiology (MOOSE) (7), with the following questions: What are the vitamin E concentrations in different periods of lactation of healthy breast milk? Do the vitamin E concentrations in breast milk of normal mothers vary in different regions?

### Search strategy

Studies published before May 2021 were searched by three independent reviewers in databases of CNKI (Chinese), WanFang Data (Chinese), VIP (Chinese), PubMed, Cochrane Library, Web of Science and Embase. The following key words were used (Table 1). Authors were contacted when full-text of articles were not available.

**Table 1:** Literature search.

**Databases**	**Key words**
CNKI/WanFang Data/VIP	The research strategy of Chinese databases adopted included different combinations of the following terms in Chinese: “Vitamin E”, “tocopherol”, “breast milk”, “mother milk”, “breastfeed”.
PubMed/Cochrane Library	(‘Vitamin E'*[Title/Abstract] OR tocopherol[Title/Abstract]) AND (‘breast milk'[Title/Abstract] OR ‘breast* milk'[Title/Abstract] OR ‘human* milk'[Title/Abstract] OR ‘mother* milk'[Title/Abstract] OR ‘woman* milk'[Title/Abstract] OR ‘women* milk'[Title/Abstract] OR ((lactating OR lactation) AND milk)[Title/Abstract])
	(‘Vitamin E'*[Title/Abstract] OR tocopherol[Title/Abstract]) AND (breastfed[Title/Abstract] OR breastfeed[Title/Abstract] OR breastfeeding[Title/Abstract] OR ‘breast fed'[Title/Abstract] OR ‘breast feed'[Title/Abstract] OR ‘breast feeding'[Title/Abstract])
Web of Science	((TS=(tocopherol)) OR TS=(vitamin E)) AND ((((((TS=(breastfeeding)) OR TS=(breastfed)) OR TS=(breast feeding)) OR TS=(breast fed)) OR TS=(breastfeed)) OR TS=(breast feed))
	((TS=(tocopherol)) OR TS=(vitamin E)) AND ((((((TS=(human* milk)) OR TS=(woman*milk)) OR TS=(mother* milk)) OR TS=(breast* milk)) OR TS=(lactation)) OR TS=(lactating))
Embase	(tocopherol*:ab,ti OR ‘vitaminE':ab,ti) AND (‘breastmilk':ab,ti OR ‘breast* milk':ab,ti OR ‘human* milk':ab,ti OR ‘mother* milk':ab,ti OR ‘woman* milk':ab,ti OR ‘women* milk':ab,ti OR ((lactating OR lactation) AND milk)) :ab,ti)
	(tocopherol*:ab,ti OR ‘vitamin E':ab,ti) AND (breastfed:ab,ti OR breastfeed:ab,ti OR breastfeeding:ab,ti OR ‘breast fed':ab,ti OR ‘breast feed':ab,ti OR ‘breast feeding':ab,ti)

### Eligibility criteria

We adopted as inclusion criteria the studies that:

- language was Chinese or English;
- involved lactating mothers aged from 18 to 45 years old in addition to infants aged from 0 to 48 months;
- either mothers or infants were medically certified as healthy;
- involved intervention studies and observational studies (cross-sectional study, case-control study, cohort study). Lactating mothers in control group in randomized controlled trials, who did not intake special dietary or participate in dietary supplementation, were included in this meta-analysis;
- the concentration of vitamin E in breast milk was detected by High Performance Liquid Chromatography (HPLC) or Ultra High Performance Liquid Chromatographic (UHPLC).

The studies were excluded that:

- lactating mothers were active smokers, or with chronic conditions (such as gestational diabetes or mastitis), or undergoing pharmacotherapy;
- lactating mothers received interventions from special diets or dietary supplements;
- lactation stages were not described distinctly;
- the main outcomes did not have values;
- included conference papers, reviews, ecological studies, case reports, editorials, letters, commentary, short surveys, and notes.

### Study selection and data extraction

The workflow is presented in Table 1. First, duplicate studies were removed manually or by using Endnote. Next, titles and abstracts screening were performed in order to exclude the irrelevant studies. Full-text articles which needed further investigation were assessed by eligibility criteria.

Two researchers screened information and extracted the data independently, and disagreements were resolved by consensus. When a consensus could not be reached, the third reviewer was consulted. The following information was extracted from the final included articles, which included the first author, year of publication, country, lactation stage, sample size, relevant characteristics of mother (age, gestational weeks etc.) and data of vitamin E concentration.

### Assessment of study quality

The quality of studies was assessed according to the Joanna Briggs Institute (JBI) critical appraisal checklist (8–10). This assessment tool was chosen as it has been widely used in systematic reviews.

### Statistical analysis

#### Data conversion

Total vitamin E activity was calculated as follows (11–13):


$$\begin{array}{l} \alpha -TE=\left(mg\alpha -tocopherol\times 1.0\right)+\left(mg\beta -tocopherol\times 0.5\right)\\ \;+\left(mg\gamma -tocopherol\times 0.1\right)+\left(mg\delta -tocopherol\times 0.03\right)\\ \;+\left(mg\alpha -tocotrienol\times 0.3\right)+\left(mg\;\beta -tocotrienol\times 0.05\right). \end{array}$$

The vitamin E data reported in different units were converted to mg α-TE/l uniformly. For instance, millimoles could be converted to milligrams by multiplying by molecular weight. Breast milk data used per kilogram could be converted to per liter by dividing by 1.032.

#### Data consolidation

Data in different studies presented in non-consistent forms, such as median, minimum/maximum values, and/or quartiles. Therefore, sample mean and standard deviation were estimated to pool results in a consistent format (14, 15).

If multiple data existed in the same lactation period in one study, the weighted mean (Means) and standard deviation (SDs) could be calculated with the following formula:


$$\begin{array}{l} \text{Means}\;\;=\;\frac{\left(n_{1}\times M_{1}+n_{2}\times M_{2}+n_{3}\times M_{3}+\cdots +n_{i}\times M_{i}\right)}{\left(n_{1}+n_{2}+n_{3}+\cdots +n_{i}\right)}\\ \;\;\;\;\;\;\;{\text{A}}_{\text{i}}\;\;=\;S_{i}^{\;\;2}(n_{i}-1)+M_{i}^{\;\;2}\times n_{i}\\ \;\;\;\;\text{ SDs}\;\;=\;\sqrt{\frac{{\sum^{\text{​}}}^{\text{​}}A_{i}-\frac{{\left[{\sum^{\text{​}}}^{\text{​}}(M_{i}n_{i})\right]}^{2}}{N}}{N-1}} \end{array}$$

Where: *n*_*i*_ =Sample size of individual studies, *M*_*i*_ =Mean of individual studies, *S*_*i*_ = Standard deviation of individual studies.

#### Meta-analysis

The meta-analysis was performed by Stata software (version 14.0). The program of “metan” was used to pool vitamin E concentration in the format of means with 95% confidence intervals (95% CIs). The *I*^2^ and the Cochran Q test were used to assess heterogeneity. *I*^2^ > 50% was considered to have substantial heterogeneity, and the random effect model was chosen. Otherwise, the fixed-effect model was used. The publication bias was evaluated by Egger's test and trim-and-fill analysis. *P*-value < 0.05 was considered as statistically significant. The trim-and-fill analysis was a non-parametric method for approximating the number of missing studies that might help in reducing and correcting publication bias in meta-analysis.

The possible sources of heterogeneity were identified by the multivariable meta-regression model. Subgroup analyses were conducted based on publication year, country of study, region and research type. Sensitivity analysis was also conducted to examine the effect of every study on the final results.

## Result

### Data search results and included studies

**Figure 1:**
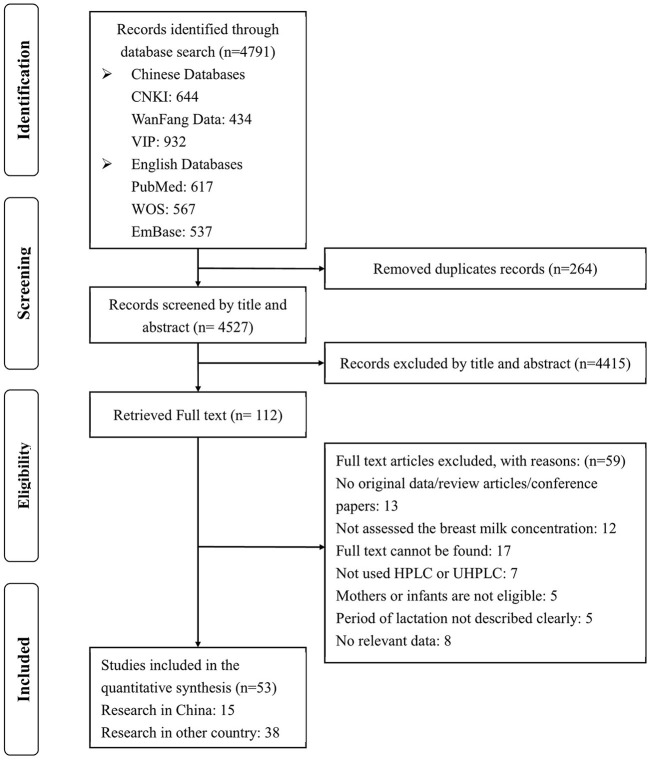
Study selection flow.

Four thousand and seven hundred and ninety one studies were found from all databases. Then, 4,527 studies were reserved after duplicates were removed and 4,415 articles were excluded by checking the titles and abstracts. For the remaining 112 articles, the full texts were rigorously reviewed. After the screening, 53 papers were included in this study (Figure 1).

### Study characteristics

Of the 53 included articles, 46 were descriptive studies (6, 16–60), 1 was a case-control study (61), 1 was a non-randomized controlled trial (62), and 5 were randomized controlled trials (63–67). A summary of these findings was presented in Table 2 and Supplementary Tables S1–S4.

**Table 2:** Characteristics of included studies for the meta-analysis.

**Reference**	**Country**	**Region**	**Type of study**	**Colostrum**		**Transitional milk**		**Mature milk**		**Study quality**
				**Sample size**	**Concentration (mg α-TE/l)**	**Sample size**	**Concentration (mg α-TE/l)**	**Sample size**	**Concentration (mg α-TE/l)**	
Lennart et al. (16)	Sweden	Western country	cross-sectional study	6	10.00 ± 5.50	10	4.80 ± 1.80	24	3.20 ± 1.80	Medium
Chappell et al. (17)	Canada	Western country	cross-sectional study	12	15.00 ± 2.50^a^					Medium
Chappell et al. (18)	Canada	Western country	longitudinal study	12	15.48 ± 8.80^a^			12	1.50 ± 6.34	Medium
Haug et al. (19)	Germany	Western country	longitudinal study	25	8.33 ± 9.82^a^			34	3.19 ± 1.35	Medium
Moffatt et al. (20)	America	Western country	cross-sectional study					5	3.12 ± 0.58	Medium
Boersma et al. (21)	Saint Lucia	Western country	longitudinal study	13	22.39 ± 14.3	11	13.59 ± 8.65	12	8.24 ± 4.8	High
Zheng et al. (47)	China	Asian country	cross-sectional study	43	6.94 ± 3.51^a^					Medium
Zheng et al. (48)	China	Asian country	cross-sectional study	38	3.45 ± 1.18^a^	5	1.32 ± 0.59^a^			Medium
Zheng et al. (Chinese) (49)	China	Asian country	cross-sectional study	71	5.57 ± 2.70					Medium
Barua et al. (22)	Bangladesh	Asian country	cross-sectional study					61	2.04 ± 0.86	High
Barbas et al. (23)	Spain	Western country	longitudinal study	8	14.40 ± 6.50			8	3.10 ± 1.40	Medium
Ortega et al. (24)	Brazil	Western country	longitudinal study			57	1.80 ± 0.68	57	0.96 ± 0.31	High
Zheng et al. (Chinese) (50)	China	Asian country	cross-sectional study	12	9.12 ± 1.40					High
Zheng et al. (Chinese) (67)	China	Asian country	randomized controlled trial	30	7.30 ± 3.29^b^					Medium
Macias et al. (25)	Cuba	Western country	longitudinal study	21	11.80 ± 6.30	21	5.00 ± 3.00	21	2.70 ± 1.10	High
Olafsdottir et al. (26)	Iceland	Western country	cross-sectional study					77	4.4 ± 1.85	High
Zhu et al. (Chinese) (51)	China	Asian country	longitudinal study	40	8.98 ± 3.74	40	4.47 ± 1.64	40	3.31 ± 1.13	High
Schweigert et al. (27)	Germany	Western country	longitudinal study	21	22.01 ± 13.39			21	5.69 ± 2.20	High
Sakurai et al. (28)	Japan	Asian country	cross-sectional study	6	5.95 ± 2.65	6	5.23 ± 2.67	103	2.98 ± 1.28	High
Romeu-Nadal et al. (29)	Spain	Western country	cross-sectional study					10	3.89 ± 0.16	Medium
Tokusoglu et al. (30)	Turkey	Western country	cross-sectional study					92	9.84 ± 2.13	High
Sziklai-László et al. (33)	Hungary	Western country	cross-sectional study			12	4.19 ± 2.20	18	3.12 ± 1.20	High
Grazyna et al. (31)	Poland	Western country	cross-sectional study					30	4.13 ± 1.94	High
Molto-Puigmarti et al. (32)	Spain	Western country	longitudinal study	10	37.93 ± 24.57			10	3.87 ± 2.48	Medium
Tijerina-Sáenz et al. (34)	Canada	Western country	cross-sectional study					60	2.27 ± 0.92	Medium
Orhon et al. (61)	Turkey	Western country	case-control study	20	13.27 ± 0.69^b^					High
Garcia et al. (62)	Brazil	Western country	non-randomized controlled trial	74	10.81 ± 7.42^b^					High
Yu et al. (52)	China	Asian country	cross-sectional study	7	3.04 ± 1.94	7	1.80 ± 0.62	66	2.42 ± 1.64	High
Antonakou et al. (35)	Greece	Western country	cross-sectional study					126	3.85 ± 1.86	High
Kasparova et al. (36)	Czech Republic	Western country	cross-sectional study					48	3.83 ± 1.45	Medium
Szlagatys-Sidorkiewicz et al. (6)	Poland	Western country	longitudinal study	49	8.69 ± 5.18			49	1.94 ± 2.41	High
Martysiak-Zurowska et al. (39)	Poland	Western country	longitudinal study	17	10.13 ± 1.50	30	4.59 ± 0.93	46	2.64 ± 0.89	High
de Lira et al. (37)	Brazil	Western country	cross-sectional study	103	11.24 ± 5.51					High
Grilo et al. (38)	Brazil	Western country	cross-sectional study	71	10.94 ± 5.32^a^					High
Fang et al. (Chinese) (53)	China	Asian country	cross-sectional study	72	9.29 ± 5.33			31	2.90 ± 1.50	Medium
Clemente et al. (63)	Brazil	Western country	randomized controlled trial	72	16.54 ± 1.71^b^					High
Liu et al. (Chinese) (55)	China	Asian country	cross-sectional study	5	2.13 ± 0.91	10	2.21 ± 1.12	38	2.49 ± 1.01	High
Jiang et al. (54)	China	Asian country	longitudinal study	102	6.32 ± 4.25	102	2.56 ± 2.25	102	1.83 ± 1.12	High
Grilo et al. (64)	Brazil	Western country	randomized controlled trial	88	12.02 ± 6.78^b^			27	2.48 ± 1.01	Medium
Xue et al. (56)	China	Asian country	cross-sectional study	77	7.76 ± 6.13	89	4.25 ± 2.56	270	2.70 ± 1.78	High
Kim et al. (40)	Korea	Asian country	cross-sectional study					165	2.10 ± 1.10	High
Silva et al. (42)	Brazil	Western country	cross-sectional study	100	17.44 ± 6.46	77	5.99 ± 2.24	63	3.45 ± 1.64	High
Melo et al. (65)	Brazil	Western country	randomized controlled trial	78	15.80 ± 8.83^b^					Medium
Samano et al. (41)	Mexico	Western country	cross-sectional study					32	6.64 ± 3.2	High
Wei et al. (57)	China	Asian country	longitudinal study	103	7.50 ± 2.10	103	3.80 ± 1.40	103	3.10 ± 1.40	High
Wu et al. (Chinese) (58)	China	Asian country	longitudinal study	89	11.81 ± 5.33	89	4.69 ± 1.81	89	4.26 ± 2.05	High
de Sousa Reboucas et al. (66)	Brazil	Western country	randomized controlled trial					80	2.98 ± 0.81^b^	Medium
Machado et al. (43)	Brazil	Western country	cross-sectional study					38	0.52 ± 0.10	High
da Mata et al. (44)	Brazil	Western country	cross-sectional study					103	3.06 ± 1.70	High
Wu et al. (Chinese) (60)	China	Asian country	longitudinal study	89	9.72 ± 5.22	89	4.58 ± 1.81	89	4.23 ± 1.95	High
Wu et al. (59)	China	Asian country	longitudinal study	42	10.12 ± 4.52	42	5.35 ± 1.97	42	3.73 ± 1.63	High
Duan et al. (45)	Korea	Asian country	cross-sectional study					34	3.82 ± 1.75	High
Zagierski et al. (46)	Poland	Western country	cross-sectional study					154	3.82 ± 1.22	High

a Data refer to healthy mothers of full-term infants.

b Data refer only to the control group.

It should be noticed that 7 studies reported vitamin E concentrations in breast milk from mothers of preterm and full-term infants both. However, the results of normal mothers who gave birth to full-term infants were used only in present study. Moreover, the data of vitamin E concentrations in healthy control group were chosen in case-control study, non-randomized controlled or randomized controlled trials.

### Meta-analysis results

#### Results of syntheses

The pooled mean vitamin E concentration in colostrum was 10.57 mg α-TE/L (95%CI 8.94–12.20), transitional milk was 4.03 mg α-TE/L (95%CI 3.29–4.77), and mature milk was 3.29 mg α-TE/L (95%CI 2.95–3.64) (Table 3).

**Table 3:** Meta-analysis summary.

**Periods of lactation**	**Concentration of vitamin E**					
	**Number of studies**	**Sample size**	**Overall effect (95% CI)**	**Heterogeneity test**		**Egger's test**
				***I^2^* (%)**	***P***	***P***
Colostrum milk	35	1,626	10.57 (8.94–12.20)	99.1	0.000	0.954
Transitional milk	18	800	4.03 (3.29–4.77)	97.8	0.000	0.063
Mature milk	42	2,562	3.39 (2.86–3.92)	99.6	0.000	0.000

**Figure 2:**
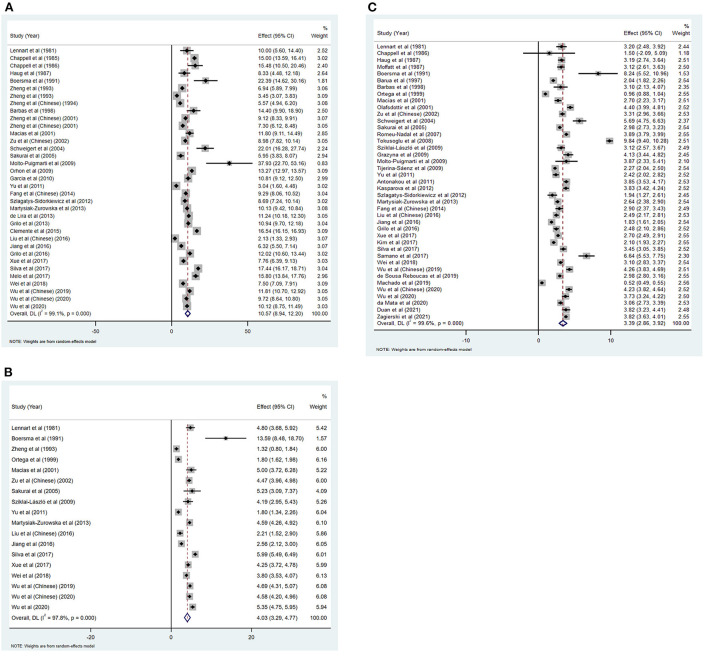
Forest plot of vitamin E concentration in colostrum **(A)**, transitional milk **(B)** and mature milk **(C)**.

Forest plot showed that the minimum and maximum values of vitamin E content in colostrum were 2.13 mg α-TE/L (55) and 37.93 mg α-TE/L (32), in transitional milk were 1.32 mg α-TE/L (48) and 13.59 mg α-TE/L (21), in mature were 0.52 mg α-TE/L (43) and 9.84 mg α-TE/L (30) (Figure 2).

#### Heterogeneity

Publication year, region (Asia or not), country of study and research type were analyzed for the source of heterogeneity by meta-regression analysis (multivariable). Results showed region might be the source of heterogeneity in colostrum (Table 4). We provide summary estimates of vitamin E content; however, the *I*^2^ statistic indicated that data were heterogeneous in many of our analyses and therefore these summary measures must be interpreted with appropriate caution.

**Table 4:** Meta-analysis summary.

**Periods of lactation**	**Publication year**		**Country**		**Region**		**Research type**	
	**Coefficient**	***P***	**Coefficient**	***P***	**Coefficient**	***P***	**Coefficient**	***P***
Colostrum milk	0.079	0.200	−0.355	0.049	8.821	0.000	0.281	0.562
Transitional milk	0.023	0.679	−0.023	0.861	1.656	0.367	0.648	0.530
Mature milk	0.004	0.888	0.013	0.826	0.613	0.414	−0.217	0.477

#### Subgroup analyses

The pooled concentration of vitamin E in colostrum was 13.34 mg α-TE/L (95%CI 11.97–14.72) in Western countries (19 studies were included) and 7.18 mg α-TE/L (95%CI 5.84–8.52) in Asian countries (16 studies were included).

**Figure 3:**
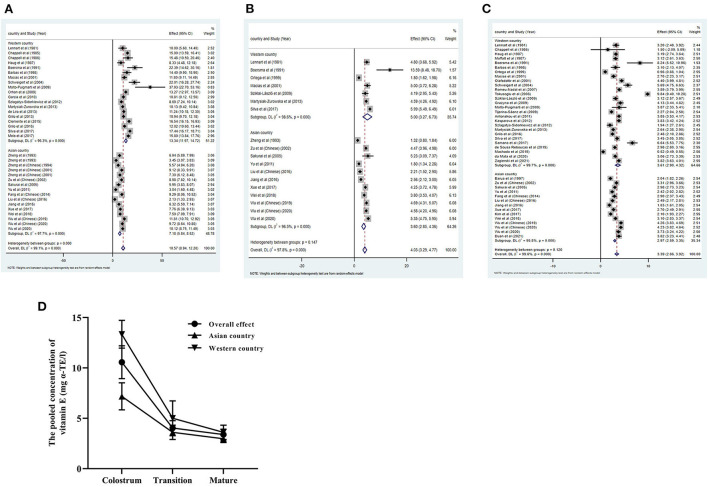
Sub-group analysis showed the pooled concentration of vitamin E based on Western countries and Asian countries in colostrum **(A)**, transitional milk **(B)** and mature milk **(C)**. The trend of pooled results in different periods of lactation **(D)**.

The result in transitional milk was 5.00 mg α-TE/L (95%CI 3.27–6.73) in Western countries (7 studies were included) and 3.61 mg α-TE/L (95%CI 2.90–4.32) in Asian countries (11 studies were included). The data in mature milk was 3.61 mg α-TE/L (95%CI 2.90–4.32) in Western countries (29 studies were included) and 2.97 mg α-TE/L (95%CI 2.59–3.35) in Asian countries (14 studies were included) (Figure 3).

#### Sensitivity analyses

**Figure 4:**
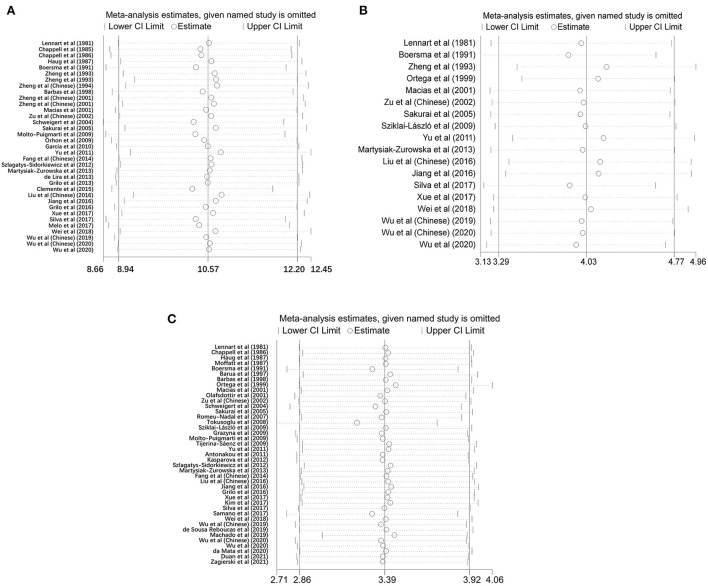
Sensitivity analysis showed the influential studies on the overall pooled effect for colostrum **(A)**, transitional milk **(B)** and mature milk **(C)**.

In this review, most studies had the consistent influence on the overall estimation of meta-analysis except three articles (21, 30, 43), which had a small influence over other researches of mature milk (Figure 4).

#### Publication bias

**Figure 5:**
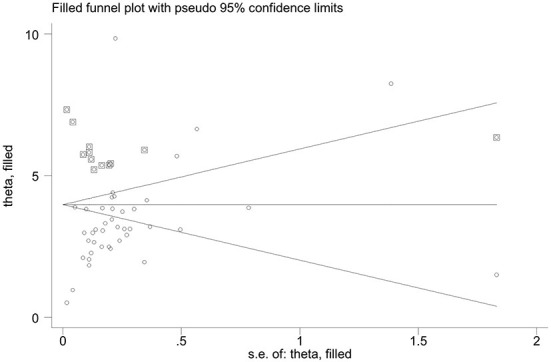
Trim-and-fill analysis estimated the number of possible missing studies for the vitamin E concentration in mature milk. The squares represented the possible missing studies.

The Egger's test of mature milk (*P* < 0.001) revealed evidence of publication bias. Trim-and-fill analysis estimated 12 missing studies. The overall effect measure based on this analysis was 3.98 mg α-TE/L (95%CI 3.02–4.93) (Figure 5), which was slightly higher than the originally reported overall effect measure (Figure 2C). This adjusted estimate suggested a lower risk of bias than the original analysis.

## Discussion

To our knowledge, this could be the first meta-analysis that evaluated the level of vitamin E in healthy mothers at different stages of lactation and it revealed a number of interesting findings.

### Colostrum milk

Colostrum, which is generated from the first day until the seventh or tenth day following parturition, is the first milk lactated (68). The publication of World Health Organization (WHO) and United Nations Children's Fund (?UNICEF)? have demonstrated that breastfeeding with colostrum milk within the first hour of new life could effectively decrease neonatal mortality. It undoubtedly highlights the significance of breastfeeding right away upon delivery (69).

It is reported that colostrum is characterized by the highest concentration of vitamin E. The significant reduction can be observed in transitional milk and mature milk. Given that the concentration of vitamin E in plasma of neonates is usually much lower than that of adults including their mothers, high vitamin E consumption from colostrum seems to provide a compensatory mechanism of antioxidative activity (6).

In this study, 35 evidence demonstrated the level of vitamin E in colostrum, 18 and 42 evidence reported the vitamin E concentration in transitional milk and mature milk, respectively. The results of the meta-analysis showed vitamin E concentration was significantly higher in colostrum (10.57 mg α-TE/L) than in transitional milk (4.03 mg α-TE/L) and mature milk (3.29 mg α-TE/L). The trend of these pooled results was in line with the longitudinal studies that reported different lactation periods (16, 21, 25, 28, 39, 42, 51, 54, 56–60). Throughout lactation, vitamin E levels decreased constantly. This vitamin E reduction in breast milk could be explained by the fact that, after the first few days of lactation, the diameter of milk fat globules increases as milk matures, and the synthesis and secretion of triglycerides increase, without a proportional increase in the secretion of phospholipids and other components (including tocopherols, cholesterol, and the percentage of long-chain PUFAs) of the membranes of fat globules (70). Therefore, there is a significant reduction in the levels of alpha-tocopherol.

The studies that were chosen included 19 researches on the colostrum of Western lactating women and 16 studies of Asian lactating women. Subgroup analyses showed that Asian women had significantly lower levels of vitamin E in their colostrum than did Western women. The reason for this difference might be discovered through comparing results between original studies. Maternal characteristics, genetic background, dietary intake of vitamin E and the use of supplementation appeared to be the main factors for the discrepancy of vitamin E level in breast colostrum between different regions (2, 57, 65, 68).

It's worth noting that a discrepancy of vitamin E concentration could be observed in different research times. In recent 10 years, colostrum are explored in 8 studies in Western lactating women. The vitamin E concentrations (16.54 ± 1.71 mg/L, 12.02 ± 6.78 mg/L, 17.44 ± 6.46 mg/L, 15.8 ± 8.83 mg/L) (42, 63–65) in the latest 4 Brazilian studies from 2015 to 2017 were higher than those in the articles from Poland (8.69 ± 5.18 mg/L, 10.13 ± 1.5 mg/L) (6, 39) and Brazil (11.24 ± 5.51 mg/L, 10.94 ± 5.32 mg/L) (37, 38) both in 2013. The same phenomenon could be found in the research of China. Moreover, vitamin E levels in colostrum also could be found regional discrepancy in China (Supplementary Figure S1). Three researches of Wu et al. (58–60) observed the vitamin E values of colostrum in Shanghai (9.72 ± 5.22 mg/L, 10.12 ± 4.52 mg/L, 11.81 ± 5.33 mg/L) from 2019 to 2020 were much higher than Inner Mongolia (3.04 ± 1.94 mg/L) in 2009 (52), Hohhot (2.13 ± 0.91 mg/L) in 2013 (55), Hangzhou (4.40 ± 2.85 mg/L) in 2016 (54), Lanzhou (8.09 ± 4.85 mg/L) in 2016 (54), Beijing (6.53 ± 4.12 mg/L) in 2016 (54). The reason might be associated with the improved economic conditions and increased breastfeeding health awareness. Improving the nutritional status of breastfeeding mothers has an extremely important impact on the ideal breast milk of lactating mothers.

### Transitional milk

The composition of milk gradually changes after childbirth. Breast milk produced from the eighth to the fifteenth day after delivery was known as transitional milk (68). The vitamin E concentration in transitional milk was lower than that in colostrum but higher than that in mature milk, which is similar to other researches (16, 21, 24, 25, 28, 33, 39, 42, 51, 54, 56–60).

The subgroup analyses observed that vitamin E concentration in transitional milk of Western lactating women was higher than that of Asian lactating women. Other important factors must be taken into account in addition to dietary restrictions and ethnicity. We found that the collection time of transitional milk was inconsistent in various studies. For example, the transitional milk is collected from the 21st to the 24th day postpartum (51), or the 8th to the 21th day postpartum (52, 55) in several prior studies in China. However, the latest studies in China (54, 56–60) revealed the collection time is from the 5th to 15th day postpartum, which is comparable to the majority of studies conducted in other nations (16, 21, 24, 25, 33, 39, 42). It might be an important reason resulting in the lower pooled vitamin E concentration of transitional milk in Asian countries. In order to increase the reliability of the results, more researches of transitional milk collected from the 5th to 15th day postpartum are needed.

Moreover, in a study of Saint Lucia (21), the result of vitamin E concentration in transitional milk was much higher (approximately two to three times) than that of other Western countries. However, the author did not mention the reason for this unusually high concentration. Due to the lack of transitional milk studies, the overall effect of meta-analysis of Western countries was 4.36 (95%CI 2.62–6.14) after excluding this abnormal value. One thing worth noting is the exclusion could cause a big discrepancy in results. Therefore, more data is needed to support the values as well.

### Mature milk

After transition milk, variations in the composition of breast milk continue to occur, until third week postpartum. During this period, the composition of milk becomes more stable, which is mature milk (68).

The concentration of vitamin E in mature breast milk samples from Western countries were near to the values of Asian samples. It was speculated that individual or dietary factors might have little influence on mature breast milk. This speculation was supported by a study, which demonstrated maternal supplementation with R, R, R, α-tocopherol could increase vitamin E level of colostrum and transitional milk rather than mature milk (71). It is worth noting that infants with an estimated daily intake of 780 mL/day may not get enough vitamin E from mature milk to meet their nutritional needs (42, 56). Consequently, the implementation of procedures to increase the level of vitamin E in milk would be important especially for nursing mothers living in poor conditions of food safety.

Furthermore, Xue et al. (56) study the vitamin E concentration of breast milk during 12–240 day postpartum. It was found that the concentration of vitamin E in breast milk observed in 12–30th day postpartum (2.96 ± 2.11 mg/L) were similar to those collected in 31th-240th day postpartum (31–60th day: 2.96 ± 1.92 mg/L, 61–120th day: 2.45 ± 1.67 mg/L, 121–240th day: 2.71 ± 1.72 mg/L). It could be implied that vitamin E concentration in breast milk might reach a relatively stable level after 12th day postpartum. Another study observed in Japan in 2005 (28) showed that vitamin E concentration in breast milk in 21–89th day postpartum (2.97 ± 1.23 mg α-TE/L) were same as those in 90–180th day and 181–365th day postpartum (3.45 ± 1.39 mg α-TE/L and 2.52 ± 1.03 mg α-TE/L). The plateau of vitamin E concentration in breast milk appeared almost one week later than the result of Xue et al. It is speculated that the vitamin E of breast milk continues to decrease after childbirth, until approximately second to third week postpartum. The vitamin E concentration in mature milk becomes more stable. However, to support the start of the vitamin E stationary phase, more evidence should be done.

One Turkish study in 2008 (30) showed that the content of vitamin E in mature milk (9.84 ± 2.13mg/L) was significantly greater (more than three times) than the samples from other nations such as Greece (35) and Spain (32). As a result, the statistical data of this paper may be influenced by the potential confounders. According to a Brazil study in 2019, the α-tocopherol content of breast milk was only 0.56 ± 0.11mg/L from 17th to 28th days postpartum, significantly lower than other studies conducted there during the same time period (44, 66). It may be associated with lower sample size.

### Limitation

There were some limitations to our study. First, the search was restricted to the studies published in English language and Chinese language, which may lead to publication bias. However, we have addressed the issue of publication bias during our analysis. Next, although subgroup and sensitivity analyses were performed, heterogeneity was still very large in the meta-analysis. Except for differences in region may lead to greater heterogeneity between studies especially in colostrum, the other factors could also contribute to heterogeneity. The underlying factors, including milk sample collection method, different techniques for nutrient measurements, postpartum milk sampling, time of milk sampling, duration of breastfeeding and so forth, might partly explain the large variation between studies in different periods of lactation. Therefore, more studies are necessary for reliable results.

## Conclusion

Vitamin E concentration in breast milk decreased during lactation until the mature milk was produced. The higher value of vitamin E in colostrum might be important for new-borns to defend early oxidative stress. The vitamin E concentration in colostrum from western countries was higher than from Asia, which might be related to dietary habits, individual variation, etc. More evidences of vitamin E concentration in transitional milk, especially the milk collected from the 5 to 15th day postpartum, are needed. The vitamin E content of mature milk was similar. It tended to be stable from about second week postpartum to 4–6th month postpartum. More results are needed to support this conclusion.

## Data availability statement

The original contributions presented in the study are included in the article/Supplementary material, further inquiries can be directed to the corresponding authors.

## Author contributions

YX, HZ, and AZ: applied the literature search and undertook the screening title and abstract screening. XW and XR: extracted the data and tabulated results. KL, YX, and XW: statistical analysis. YX and XW: wrote the initial version of the manuscript. YY: validation of the paper for important figures. JL and RX: critical revision of the paper for important intellectual content. All authors contributed to its final version and read and approved the final manuscript.

## Funding

This research was funded by the CNS Research Fund for DRIs; the National Natural Science Foundation of China (82273620, 81973018, and 82003459); the Key Laboratory of Trace Element and Nutrition, National Health Commission of China (WLKFZ202201).

## Conflict of interest

The authors declare that the research was conducted in the absence of any commercial or financial relationships that could be construed as a potential conflict of interest.

## Publisher's note

All claims expressed in this article are solely those of the authors and do not necessarily represent those of their affiliated organizations, or those of the publisher, the editors and the reviewers. Any product that may be evaluated in this article, or claim that may be made by its manufacturer, is not guaranteed or endorsed by the publisher.

## References

[1] Innis SM. Impact of maternal diet on human milk composition and neurological development of infants. Am J Clin Nutr. (2014) 99:734S−41S. 10.3945/ajcn.113.072595 24500153

[2] Keikha M Shayan-Moghadam R Bahreynian M Kelishadi R. Nutritional supplements and mother's milk composition: a systematic review of interventional studies. Int Breastfeed J. (2021) 16:1. 10.1186/s13006-020-00354-0 33397426 PMC7780633

[3] Azzi A. Many tocopherols, one vitamin E. Mol Aspects Med. (2018) 61:92–103. 10.1016/j.mam.2017.06.004 28624327

[4] Mustacich DJ Bruno RS Traber MG. Vitamin E. Vitam Horm. (2007) 76:1–21. 10.1016/S0083-6729(07)76001-6 17628169

[5] Arai H Kono N. α-Tocopherol transfer protein (α-TTP). Free Radic Biol Med. (2021) 176:162–75. 10.1016/j.freeradbiomed.2021.09.021 34563650

[6] Szlagatys-Sidorkiewicz A Zagierski M Jankowska A Luczak G Macur K Baczek T . Longitudinal study of vitamins A, E and lipid oxidative damage in human milk throughout lactation. Early Hum Dev. (2012) 88:421–4. 10.1016/j.earlhumdev.2011.10.007 22085741

[7] Stroup DF Berlin JA Morton SC Olkin I Williamson GD Rennie D . Meta-analysis of observational studies in epidemiology: a proposal for reporting. Meta-analysis Of Observational Studies in Epidemiology (MOOSE) group. JAMA. (2000) 283:2008–12. 10.1001/jama.283.15.2008 10789670

[8] Tufanaru C Munn Z Aromataris E Campbell J Hopp L. Chapter 3: Systematic reviews of effectiveness. In: Aromataris E Munn Z, editors. JBI Manual for Evidence Synthesis. JBI. (2020). p. 71-133. Available online at: https://synthesismanual.jbi.global (accessed July 15, 2022).

[9] Moola S Munn Z Tufanaru C Aromataris E Sears K Sfetcu R . Chapter 7: Systematic reviews of etiology and risk. In:Aromataris E Munn Z, editors. JBI Manual for Evidence Synthesis. JBI. (2020). p. 217-69. Available from https://synthesismanual.jbi.global (accessed July 15, 2022).

[10] Munn Z Moola S Lisy K Riitano D Tufanaru C. Methodological guidance for systematic reviews of observational epidemiological studies reporting prevalence and cumulative incidence data. Int J Evid Based Healthc. (2015) 13:147–53. 10.1097/XEB.0000000000000054 26317388

[11] National Coordinating Committee on Food and Nutrition (NCCFN). Recommended Nutrient Intakes for Malaysia. Putrajaya: Ministry of Health Malaysia; (2017).

[12] Institute of Medicine. Dietary Reference Intakes for Vitamin C, Vitamin E, Selenium, and Carotenoids. Washington, DC: The National Academies Press (2000). Available online at: 10.17226/9810 (accessed July 15, 2022). 25077263

[13] Chinese Nutrition Society (CNS). Chinese Dietary Reference Intakes. Beijing: Science Press (2014).

[14] Wan X Wang W Liu J Tong T. Estimating the sample mean and standard deviation from the sample size, median, range and/or interquartile range. BMC Med Res Methodol. (2014) 14:135. 10.1186/1471-2288-14-135 25524443 PMC4383202

[15] Higgins JPT Thomas J Chandler J Cumpston M Li T Page MJ . Cochrane Handbook for Systematic Reviews of Interventions version 6.2 (updated February 2021). Cochrane. (2021). New York, NY: John Wiley & Sons. Available from www.training.cochrane.org/handbook (accessed July 15, 2022).

[16] Jansson L Akesson B Holmberg L. Vitamin E and fatty acid composition of human milk. Am J Clin Nutr. (1981) 34:8–13. 10.1093/ajcn/34.1.8 7446463

[17] Chappell JE Francis T Clandinin MT. Vitamin A and E content of human milk at early stages of lactation. Early Hum Dev. (1985) 11:157–67. 10.1016/0378-3782(85)90103-3 4029052

[18] Chappell JE Francis T Clandinin MT. Simultaneous high performance liquid chromatography analysis of retinol ester and tocopherol isomers in human milk. Nutr Res. (1986) 6:849–52. 10.1016/S0271-5317(86)80167-1

[19] Haug M Laubach C Burke M Harzer G. Vitamin E in human milk from mothers of preterm and term infants. J Pediatr Gastroenterol Nutr. (1987) 6:605–9. 10.1097/00005176-198707000-00020 3430268

[20] Moffatt PA Lammi-Keefe CJ Ferris AM Jensen RG. Alpha and gamma tocopherols in pooled mature human milk after storage. J Pediatr Gastroenterol Nutr. (1987) 6:225–7. 10.1097/00005176-198703000-00011 3694347

[21] Boersma ER Offringa PJ Muskiet FA Chase WM Simmons IJ. Vitamin E, lipid fractions, and fatty acid composition of colostrum, transitional milk, and mature milk: an international comparative study. Am J Clin Nutr. (1991) 53:1197–204. 10.1093/ajcn/53.5.1197 2021129

[22] Barua S Tarannum S Nahar L Mohiduzzaman M. Retinol and alpha-tocopherol content in breast milk of Bangladeshi mothers under low socio-economic status. Int J Food Sci Nutr. (1997) 48:13–8. 10.3109/09637489709006959 9093545

[23] Barbas C Herrera E. Lipid composition and vitamin E content in human colostrum and mature milk. J Physiol Biochem. (1998) 54:167–73. 10217214

[24] Ortega RM López-Sobaler AM Andrés P Martínez RM Quintas ME Requejo AM. Maternal vitamin E status during the third trimester of pregnancy in Spanish women: Influence on breast milk vitamin E concentration. Nutr Res. (1999) 19:25–36. 10.1016/S0271-5317(98)00176-69734745

[25] Macias C Schweigert FJ. Changes in the concentration of carotenoids, vitamin A, alpha-tocopherol and total lipids in human milk throughout early lactation. Ann Nutr Metab. (2001) 45:82–5. 10.1159/000046711 11359034

[26] Olafsdottir AS Wagner KH Thorsdottir I Elmadfa I. Fat-soluble vitamins in the maternal diet, influence of cod liver oil supplementation and impact of the maternal diet on human milk composition. Ann Nutr Metab. (2001) 45:265–72. 10.1159/000046737 11786649

[27] Schweigert FJ Bathe K Chen F Buscher U Dudenhausen JW. Effect of the stage of lactation in humans on carotenoid levels in milk, blood plasma and plasma lipoprotein fractions. Eur J Nutr. (2004) 43:39–44. 10.1007/s00394-004-0439-5 14991268

[28] Sakurai T Furukawa M Asoh M Kanno T Kojima T Yonekubo A. Fat-soluble and water-soluble vitamin contents of breast milk from Japanese women. J Nutr Sci Vitaminol (Tokyo). (2005) 51:239–47. 10.3177/jnsv.51.239 16261995

[29] Romeu-Nadal M Castellote AI López-Sabater MC. Effect of cold storage on vitamins C and E and fatty acids in human milk. Food Chem. (2007) 106:65–70. 10.1016/j.foodchem.2007.05.046

[30] Tokusoglu O Tansug N Aksit S Dinc G Kasirga E Ozcan C. Retinol and alpha-tocopherol concentrations in breast milk of Turkish lactating mothers under different socio-economic status. Int J Food Sci Nutr. (2008) 59:166–74. 10.1080/02699200701539171 17852471

[31] Duda G Nogala-Kalucka M Karwowska W Kupczyk B. Influence of the lactating women diet on the concentration of the lipophilic vitamins in human milk. Pakistan J Nutr. (2009) 8:629–34. 10.3923/pjn.2009.629.634

[32] Molto-Puigmarti C Castellote AI Lopez-Sabater MC. Ultra-High-Pressure Liquid Chromatographic method for the analysis of tocopherols in human colostrum and milk. J Chromatogr A. (2009) 1216:4388–94. 10.1016/j.chroma.2009.02.088 19368930

[33] Sziklai-László I Majchrzak D Elmadfa I Cser MÁ. Selenium and vitamin E concentrations in human milk and formula milk from Hungary. J Radioanal Nucl Chem. (2009) 279:585–90. 10.1007/s10967-008-7311-7

[34] Tijerina-Saenz A Innis SM Kitts DD. Antioxidant capacity of human milk and its association with vitamins A and E and fatty acid composition. Acta Paediatr. (2009) 98:1793–8. 10.1111/j.1651-2227.2009.01437.x 19807706 PMC2773529

[35] Antonakou A Chiou A Andrikopoulos NK Bakoula C Matalas AL. Breast milk tocopherol content during the first six months in exclusively breastfeeding Greek women. Eur J Nutr. (2011) 50:195–202. 10.1007/s00394-010-0129-4 20721564

[36] Kasparova M Plisek J Solichova D Krcmova L Kucerova B Hronek M . Rapid sample preparation procedure for determination of retinol and alpha-tocopherol in human breast milk. Talanta. (2012) 93:147–52. 10.1016/j.talanta.2012.01.065 22483891

[37] de Lira LQ Lima MS de Medeiros JM da Silva IF Dimenstein R. Correlation of vitamin A nutritional status on alpha-tocopherol in the colostrum of lactating women. Matern Child Nutr. (2013) 9:31–40. 10.1111/j.1740-8709.2011.00376.x 22099335 PMC6860850

[38] Grilo EC Lira LQ Dimenstein R Ribeiro KD. Influence of prematurity and birth weight on the concentration of alpha-tocopherol in colostrum milk. Rev Paul Pediatr. (2013) 31:473–9. 10.1590/S0103-05822013000400009 24473952 PMC4183043

[39] Martysiak-Zurowska D Szlagatys-Sidorkiewicz A Zagierski M. Concentrations of alpha- and gamma-tocopherols in human breast milk during the first months of lactation and in infant formulas. Matern Child Nutr. (2013) 9:473–82. 10.1111/j.1740-8709.2012.00401.x 22513202 PMC6860560

[40] Kim H Jung BM Lee BN Kim YJ Jung JA Chang N. Retinol, alpha-tocopherol, and selected minerals in breast milk of lactating women with full-term infants in South Korea. Nutr Res Pract. (2017) 11:64–9. 10.4162/nrp.2017.11.1.64 28194267 PMC5300949

[41] Samano R Martinez-Rojano H Hernandez RM Ramirez C Flores Quijano ME Espindola-Polis JM . Retinol and alpha-Tocopherol in the Breast Milk of Women after a High-Risk Pregnancy. Nutrients. (2017) 9:14. 10.3390/nu9010014 28045436 PMC5295058

[42] Silva ALC Melo LRMd Bezerra DF Queiroz JLCd Lima MSR . Vitamin E in human milk and its relation to the nutritional requirement of the term newborn. Revista Paulista de Pediatria. (2017) 35:158–64. 10.1590/1984-0462/;2017;35;2;00015 28977333 PMC5496727

[43] Machado MR Kamp F Nunes JC El-Bacha T Torres AG. Breast milk content of vitamin A and E from early- to mid-lactation is affected by inadequate dietary intake in brazilian adult women. Nutrients. (2019) 11:2025. 10.3390/nu11092025 31470574 PMC6770016

[44] da Mata AMB da Silva A Medeiros JFP Lima MSR Bezerra DS da Silva AB . Dietary Lipid Intake Influences the Alpha-tocopherol Levels in Human Milk. J Pediatr Gastroenterol Nutr. (2020) 70:858–63. 10.1097/MPG.0000000000002668 32443047

[45] Duan B So H-J Shin J-A Qin Y Yang J Lee K-T. Different content of cholesterol, retinol, and tocopherols in human milk according to its fat content. Eur Food Res Technol. (2021) 247:1307–18. 10.1007/s00217-021-03710-4

[46] Zagierski M Krukowska A Kawska K Sznurkowska K Martysiak-Zurowska D Szlagatys-Sidorkiewicz A. No evidence for sex-specificity in vitamins C, E, and fatty acid content of human milk from healthy polish mothers. J Pediatr Gastroenterol Nutr. (2021) 73:e20–e5. 10.1097/MPG.0000000000003136 33783401

[47] Zheng MC Zhang GF Zhou LS Guo XG Quan YF. Alpha-tocopherol concentrations in human milk from mothers of preterm and full-term infants in China. Biomed Environ Sci. (1993) 6:259–64. 8292270

[48] Zheng MC Zhou LS Zhang GF. Alpha-tocopherol content of breast milk in China. J Nutr Sci Vitaminol (Tokyo). (1993) 39:517–20. 10.3177/jnsv.39.517 8120675

[49] Zheng MC Zhou LS Zhang GF Liang MX. Comparison of α-tocopherol Content in Human Colostrum from Mothers Living in the City and the Rural Minority Areas. Acta Nutr Sin. (1994) 16:99-100.

[50] Zheng MC Quan YF Chen ZY Wang Y Zhang GF. Investigation of vitamin E content and its correlation in maternal blood-umbilical cord blood and breast milk. Chin J Contemp Pediatr. (2001) 3:305–6.

[51] Zhu CL Tong XB Zhang XH Zhang WY Zhao SX Hou WM . Study of vitamin E level in different phases of breast milk. Chin J Pract Pediatr. (2002) 17:624–5.

[52] Shi YD Sun GQ Zhang ZG Deng X Kang XH Liu ZD . The chemical composition of human milk from Inner Mongolia of China. Food Chem. (2011) 127:1193–8. 10.1016/j.foodchem.2011.01.123 25214113

[53] Fang F Li T Li YJ Liu B Ye WH. Investigation of the Contents of the Fat-Soluble Vitamins A, D and E in Human Milk from Hohhot. J Dairy Sci Technol. (2014) 37:5–7.

[54] Jiang J Xiao H Wu K Yu Z Ren Y Zhao Y . Retinol and alpha-tocopherol in human milk and their relationship with dietary intake during lactation. Food Funct. (2016) 7:1985–91. 10.1039/C5FO01293G 26987293

[55] Liu J. Study on the Vitamin Contents of Human Milk in Huhhot. Food Res. Dev. (2016),37:20–2.

[56] Xue Y Campos-Gimenez E Redeuil KM Leveques A Actis-Goretta L Vinyes-Pares G . Concentrations of carotenoids and tocopherols in breast milk from urban chinese mothers and their associations with maternal characteristics: a cross-sectional study. Nutrients. (2017) 9:1229. 10.3390/nu9111229 29120377 PMC5707701

[57] Wei W Yang J Xia Y Chang C Sun C Yu R . Tocopherols in human milk: Change during lactation, stability during frozen storage, and impact of maternal diet. Int Dairy J. (2018) 84:1–5. 10.1016/j.idairyj.2018.03.009

[58] Wu Ke Sun HX Mao YY Tian Fang Cai XK Zhao YR Cai MQ. Natural RRR-α-tocopherol and Syntheticα-tocopherol Stereosiomers in Human Breast Milk. Acta Nutr Sin. (2019) 41:539–43. 10.13325/j.cnki.acta.nutr.sin.2019.06.004

[59] Wu K Zhu J Zhou L Shen L Mao Y Zhao Y . Lactational changes of fatty acids and fat-soluble antioxidants in human milk from healthy Chinese mothers. Br J Nutr. (2020) 123:841–8. 10.1017/S0007114520000239 31964441

[60] Wu K Wang B Zhou LL Shen LW Cai MQ. Content of α-tocopherol and macronutrients in breast milk and associated factors. Chin J Reproduct Health. (2020) 31:414–9.31013594

[61] Orhon FS Ulukol B Kahya D Cengiz B Baskan S Tezcan S. The influence of maternal smoking on maternal and newborn oxidant and antioxidant status. Eur J Pediatr. (2009) 168:975–81. 10.1007/s00431-008-0873-0 19034508

[62] Garcia L Ribeiro K Araujo K Pires J Azevedo G Dimenstein R. Alpha-tocopherol concentration in the colostrum of nursing women supplemented with retinyl palmitate and alpha-tocopherol. J Hum Nutr Diet. (2010) 23:529–34. 10.1111/j.1365-277X.2010.01063.x 20831709

[63] Clemente HA Ramalho HM Lima MS Grilo EC Dimenstein R. Maternal supplementation with natural or synthetic vitamin E and its levels in human colostrum. J Pediatr Gastroenterol Nutr. (2015) 60:533–7. 10.1097/MPG.0000000000000635 25419678

[64] Grilo EC Medeiros WF Silva AG Gurgel CS Ramalho HM Dimenstein R. Maternal supplementation with a megadose of vitamin A reduces colostrum level of alpha-tocopherol: a randomised controlled trial. J Hum Nutr Diet. (2016) 29:652–61. 10.1111/jhn.12381 27231056

[65] Melo LRM Bezerra DF Dantas RCS Ramalho HMM Dimenstein R. Effect of maternal supplementation with vitamin E on the concentration of α-tocopherol in colostrum. Jornal de Pediatria (Versão em Português). (2017) 93:40–6. 10.1016/j.jpedp.2016.06.011 27327566

[66] de Sousa Reboucas A Costa Lemos da Silva AG Freitas de Oliveira A Thalia Pereira da Silva L de Freitas Felgueiras V Cruz MS . Factors associated with increased alpha-tocopherol content in milk in response to maternal supplementation with 800 IU of vitamin E. Nutrients. (2019) 11. 10.3390/nu11040900 31013594 PMC6520676

[67] Zheng MC Zhang DX Zhang HY Chen ZY Zhang GF. Affection of vitamin E supplementation on vitamin E concentrations of breast milk in perinatai periods. Chin J Appl Clin Pediatr. (2001) 16:43–4. 10.3969/j.issn.1003-515X.2001.01.028

[68] Lima MS Dimenstein R Ribeiro KD. Vitamin E concentration in human milk and associated factors: a literature review. J Pediatr (Rio J). (2014) 90:440–8. 10.1016/j.jped.2014.04.006 24953721

[69] World Health Organization (WHO) United Nations Children's Fund ?(UNICEF). Advocacy Strategy: Breastfeeding Advocacy Initiative, for the Best Start in Life. Geneva: World Health Organization, (2015). Report No.: WHO/NMH/NHD/15.1.

[70] Debier C. Vitamin E during pre- and postnatal periods. Vitam Horm. (2007) 76:357–73. 10.1016/S0083-6729(07)76013-2 17628181

[71] Pires Medeiros JF Ribeiro KD Lima MS das Neves RA Lima AC Dantas RC . Alpha-Tocopherol in breast milk of women with preterm delivery after a single postpartum oral dose of vitamin E. Br J Nutr. (2016) 115:1424–30. 10.1017/S0007114516000477 26931347

