# Corrigendum: Vitamin E concentration in breast milk in different periods of lactation: Meta-analysis

**DOI:** 10.3389/fnut.2022.1099704

**Published:** 2023-02-23

**Authors:** Yuandi Xi, Xianyun Wang, Kuo Liu, Huanmei Zhang, Xiangnan Ren, Ai Zhao, Yuexin Yang, Jianqiang Lai, Rong Xiao

**Affiliations:** ^1^Beijing Key Laboratory of Environmental Toxicology, School of Public Health, Capital Medical University, Beijing, China; ^2^China-DRIs Research Group on Human Milk Composition, Beijing, China; ^3^National Institute for Nutrition and Health, Chinese Center for Disease Control, Beijing, China; ^4^Key Laboratory of Human Milk Science, Chinese Center for Disease Control and Prevention, Beijing, China; ^5^Wanke School of Public Health, Tsinghua University, Beijing, China

**Keywords:** vitamin E, alpha-tocopherol, breast milk, lactation, meta-analysis

In the published article, there was an error in affiliation 2. Instead of “China-DRIs Expert Committee on Human Milk Composition, Beijing, China”, it should be “China-DRIs Research Group on Human Milk Composition, Beijing, China.”

In the published article, there was an error regarding the affiliations for Huanmei Zhang, Xiangnan Ren, and Jianqiang Lai. As well as having affiliation(s) 2, 3, they should also have Key Laboratory of Human Milk Science, Chinese Center for Disease Control and Prevention, Beijing, China.

In the published article, there was an error in the author list, and author Yuexin Yang was erroneously excluded. The corrected author list appears below.

Yuandi Xi^1,2†^, Xianyun Wang^1†^, Kuo Liu^1^, Huanmei Zhang^2,3,4^, Xiangnan Ren^2,3,4^, Ai Zhao^2,5^, Yuexin Yang^2,3^, Jianqiang Lai^2,3,4^^*^ and Rong Xiao^1^^*^

In the published article, there was an error in the Funding statement. A source of funding was missed from the statement. The correct Funding statement appears below.

