# Supplementary material for: Vitamin E concentration in breast milk in different periods of lactation: Meta-analysis
Source: Front Nutr. 2022 Nov 10;9:1050011. doi: 10.3389/fnut.2022.1050011 (PMC9691403; doi:10.3389/fnut.2022.1050011)
Supplement: Supplementary file 1 [file Data_Sheet_1.docx]

Supplementary Material

# Supplementary Tables

Supplementary Table S1. Assessment of quality of descriptive study.

| Authors | Year | Type of study | Q1 | Q2 | Q3 | Q4 | Q5 | Q6 | Q7 | Q8 | Q9 | Study  quality |
| --- | --- | --- | --- | --- | --- | --- | --- | --- | --- | --- | --- | --- |
| Lennart et al (16) | 1981 | cross-sectional study | Yes | Unclear | Unclear | No | Not applicable | Yes | Yes | Yes | Yes | Medium |
| Chappell et al (17) | 1985 | cross-sectional study | Yes | Unclear | Unclear | No | Yes | Yes | Yes | Yes | Yes | Medium |
| Chappell et al (18) | 1986 | longitudinal study | Yes | Unclear | Unclear | No | Yes | Yes | Yes | Yes | Yes | Medium |
| Haug et al (19) | 1987 | longitudinal study | Yes | Unclear | Unclear | Yes | Yes | Yes | Yes | Yes | Yes | Medium |
| Moffatt et al (20) | 1987 | cross-sectional study | Yes | Unclear | No | No | Not applicable | Yes | Yes | Yes | Yes | Medium |
| Boersma et al (21) | 1991 | longitudinal study | Yes | Yes | Yes | Yes | Not applicable | Yes | Yes | Yes | Yes | High |
| Zheng et al (47) | 1993 | cross-sectional study | Yes | Unclear | Unclear | Yes | Yes | Yes | Yes | Yes | Yes | Medium |
| Zheng et al (48) | 1993 | cross-sectional study | Yes | Unclear | Unclear | Yes | Yes | Yes | Yes | Yes | Yes | Medium |
| Zheng et al (Chinese) (49) | 1994 | cross-sectional study | Yes | Unclear | Yes | No | Not applicable | Yes | Yes | Yes | Yes | Medium |
| Barua et al (22) | 1997 | cross-sectional study | Yes | Yes | Yes | Yes | Not applicable | Yes | Yes | Yes | Yes | High |
| Barbas et al (23) | 1998 | longitudinal study | Yes | Unclear | Unclear | Yes | Not applicable | Yes | Yes | Yes | Yes | Medium |
| Ortega et al (24) | 1999 | longitudinal study | Yes | Yes | Yes | Yes | Yes | Yes | Yes | Yes | Yes | High |
| Zheng et al (Chinese) (50) | 2001 | cross-sectional study | Yes | Yes | Yes | Yes | Not applicable | Yes | Yes | Yes | Yes | High |
| Macias et al (25) | 2001 | longitudinal study | Yes | Yes | Yes | Yes | Not applicable | Yes | Yes | Yes | Yes | High |
| Olafsdottir et al (26) | 2001 | cross-sectional study | Yes | Yes | Yes | Yes | Not applicable | Yes | Yes | Yes | Yes | High |
| Zhu et al (Chinese) (51) | 2002 | longitudinal study | Yes | Yes | Yes | Yes | Not applicable | Yes | Yes | Yes | Yes | High |
| Schweigert et al (27) | 2004 | longitudinal study | Yes | Yes | Yes | Yes | Not applicable | Yes | Yes | Yes | Yes | High |
| Sakurai et al (28) | 2005 | cross-sectional study | Yes | Yes | Yes | Yes | Not applicable | Yes | Yes | Yes | Yes | High |
| Romeu-Nadal et al (29) | 2007 | cross-sectional study | Yes | Unclear | Unclear | No | Not applicable | Yes | Yes | Yes | Yes | Medium |
| Tokusoglu et al (30) | 2008 | cross-sectional study | Yes | Yes | Yes | Yes | Yes | Yes | Yes | Yes | Yes | High |
| Sziklai-László et al (33) | 2009 | cross-sectional study | Yes | Yes | Yes | No | Not applicable | Yes | Yes | Yes | Yes | High |
| Grazyna et al (31) | 2009 | cross-sectional study | Yes | Yes | Yes | Yes | Not applicable | Yes | Yes | Yes | Yes | High |
| Molto-Puigmarti et al (32) | 2009 | longitudinal study | Yes | Yes | Yes | No | Not applicable | Yes | Yes | Yes | Yes | Medium |
| Tijerina-Sáenz et al (34) | 2009 | cross-sectional study | Yes | Yes | Yes | No | Not applicable | Yes | Yes | Yes | Yes | Medium |
| Yu et al (52) | 2011 | cross-sectional study | Yes | Yes | Yes | Yes | Not applicable | Yes | Yes | Yes | Yes | High |
| Antonakou et al (35) | 2012 | cross-sectional study | Yes | Yes | Yes | Yes | Not applicable | Yes | Yes | Yes | Yes | High |
| Kasparova et al (36) | 2012 | cross-sectional study | Yes | Yes | Yes | No | Not applicable | Yes | Yes | Yes | Yes | Medium |
| Szlagatys-Sidorkiewicz et al (6) | 2012 | longitudinal study | Yes | Yes | Yes | Yes | Not applicable | Yes | Yes | Yes | Yes | High |
| Martysiak-Zurowska et al (39) | 2013 | longitudinal study | Yes | Yes | Yes | Yes | Not applicable | Yes | Yes | Yes | Yes | High |
| de Lira et al (37) | 2013 | cross-sectional study | Yes | Yes | Yes | Yes | Not applicable | Yes | Yes | Yes | Yes | High |
| Grilo et al (38) | 2013 | cross-sectional study | Yes | Yes | Yes | Yes | Yes | Yes | Yes | Yes | Yes | High |
| Fang et al (Chinese) (53) | 2014 | cross-sectional study | Yes | Unclear | Yes | No | Not applicable | Yes | Yes | Yes | Yes | Medium |
| Liu et al (Chinese) (55) | 2016 | cross-sectional study | Yes | Yes | Yes | Yes | Not applicable | Yes | Yes | Yes | Yes | High |
| Jiang et al (54) | 2016 | longitudinal study | Yes | Yes | Yes | Yes | Not applicable | Yes | Yes | Yes | Yes | High |
| Xue et al (56) | 2017 | cross-sectional study | Yes | Yes | Yes | Yes | Not applicable | Yes | Yes | Yes | Yes | High |
| Kim et al (40) | 2017 | cross-sectional study | Yes | Yes | Yes | Yes | Not applicable | Yes | Yes | Yes | Yes | High |
| Silva et al (42) | 2017 | cross-sectional study | Yes | Yes | Yes | Yes | Not applicable | Yes | Yes | Yes | Yes | High |
| Samano et al (41) | 2017 | cross-sectional study | Yes | Yes | Yes | Yes | Yes | Yes | Yes | Yes | Yes | High |
| Wei et al (57) | 2018 | longitudinal study | Yes | Yes | Yes | Yes | Not applicable | Yes | Yes | Yes | Yes | High |
| Wu et al (Chinese) (58) | 2019 | longitudinal study | Yes | Yes | Yes | Yes | Not applicable | Yes | Yes | Yes | Yes | High |
| Machado et al (43) | 2019 | cross-sectional study | Yes | Yes | Yes | Yes | Not applicable | Yes | Yes | Yes | Yes | High |
| da Mata et al (44) | 2020 | cross-sectional study | Yes | Yes | Yes | Yes | Not applicable | Yes | Yes | Yes | Yes | High |
| Wu et al (Chinese) (60) | 2020 | longitudinal study | Yes | Yes | Yes | Yes | Not applicable | Yes | Yes | Yes | Yes | High |
| Wu et al (59) | 2020 | longitudinal study | Yes | Yes | Yes | Yes | Not applicable | Yes | Yes | Yes | Yes | High |
| Duan et al (45) | 2020 | cross-sectional study | Yes | Yes | Yes | No | Not applicable | Yes | Yes | Yes | Yes | High |
| Zagierski et al (46) | 2020 | cross-sectional study | Yes | Yes | Yes | Yes | Yes | Yes | Yes | Yes | Yes | High |

Q1. Was the sample frame appropriate to address the target population?

Q2. Were study participants sampled in an appropriate way?

Q3. Was the sample size adequate?

Q4. Were the study subjects and the setting described in detail?

Q5. Was the data analysis conducted with sufficient coverage of the identified sample?

Q6. Were valid methods used for the identification of the condition?

Q7. Was the condition measured in a standard, reliable way for all participants?

Q8. Was there appropriate statistical analysis?

Q9. Was the response rate adequate, and if not, was the low response rate managed appropriately?

Supplementary Table S2. Assessment of quality of case-control study.

| Authors | Year | Type of study | Q1 | Q2 | Q3 | Q4 | Q5 | Q6 | Q7 | Q8 | Q9 | Q10 | Study  quality |
| --- | --- | --- | --- | --- | --- | --- | --- | --- | --- | --- | --- | --- | --- |
| Orhon et al. | 2010 | case-control study | Yes | Yes | Yes | Yes | Yes | No | Not applicable | Yes | Unclear | Yes | High |

Q1. Were the groups comparable other than the presence of disease in cases or the absence of disease in controls?

Q2. Were cases and controls matched appropriately?

Q3. Were the same criteria used for identification of cases and controls?

Q4. Was exposure measured in a standard, valid and reliable way?

Q5. Was exposure measured in the same way for cases and controls?

Q6. Were confounding factors identified?

Q7. Were strategies to deal with confounding factors stated?

Q8. Were outcomes assessed in a standard, valid and reliable way for cases and controls?

Q9. Was the exposure period of interest long enough to be meaningful?

Q10. Was appropriate statistical analysis used?

Supplementary Table S3. Assessment of quality of non-randomized controlled trial.

| Authors | Year | Type of study | Q1 | Q2 | Q3 | Q4 | Q5 | Q6 | Q7 | Q8 | Q9 | Study  quality |
| --- | --- | --- | --- | --- | --- | --- | --- | --- | --- | --- | --- | --- |
| Garcia et al. | 2010 | non-randomized controlled trial | Yes | Yes | Yes | Yes | No | Yes | Yes | Yes | Yes | High |

Q1. Is it clear in the study what is the ‘cause’ and what is the ‘effect’ (i.e. there is no confusion about which variable comes first)?

Q2. Were the participants included in any comparisons similar?

Q3. Were the participants included in any comparisons receiving similar treatment/care, other than the exposure or intervention of interest?

Q4. Was there a control group?

Q5. Were there multiple measurements of the outcome both pre and post the intervention/exposure?

Q6. Was follow up complete and if not, were differences between groups in terms of their follow up adequately described and analyzed?

Q7. Were the outcomes of participants included in any comparisons measured in the same way?

Q8. Were outcomes measured in a reliable way?

Q9. Was appropriate statistical analysis used?

Supplementary Table S4. Assessment of quality of randomized controlled trial.

| Authors | Year | Type of study | Q1 | Q2 | Q3 | Q4 | Q5 | Q6 | Q7 | Q8 | Q9 | Q10 | Q11 | Q12 | Q13 | Study  quality |
| --- | --- | --- | --- | --- | --- | --- | --- | --- | --- | --- | --- | --- | --- | --- | --- | --- |
| Zheng et al (Chinese) (67) | 2001 | randomized controlled trial | Yes | Unclear | Yes | Unclear | Unclear | Unclear | Unclear | Yes | No | Yes | Yes | Yes | Yes | Medium |
| Clemente et al (63) | 2015 | randomized controlled trial | Yes | Yes | Yes | Yes | Yes | No | Yes | No | Yes | Yes | Yes | Yes | Yes | High |
| Grilo et al (64) | 2016 | randomized controlled trial | Yes | Unclear | Yes | Unclear | Unclear | Unclear | Yes | Yes | Yes | Yes | Yes | Yes | Yes | Medium |
| Melo et al (65) | 2017 | randomized controlled trial | Yes | Unclear | Yes | Unclear | Unclear | Unclear | Yes | Yes | Yes | Yes | Yes | Yes | Yes | Medium |
| de Sousa Reboucas et al (66) | 2019 | randomized controlled trial | Yes | Unclear | Yes | Yes | Unclear | Unclear | Yes | Yes | Yes | Yes | Yes | Yes | Yes | Medium |

Q1. Was true randomization used for assignment of participants to treatment groups?

Q2. Was allocation to treatment groups concealed?

Q3. Were treatment groups similar at the baseline?

Q4. Were participants blind to treatment assignment?

Q5. Were those delivering treatment blind to treatment assignment?

Q6. Were outcomes assessors blind to treatment assignment?

Q7. Were treatment groups treated identically other than the intervention of interest?

Q8. Was follow up complete and if not, were differences between groups in terms of their follow up adequately described and analyzed?

Q9. Were participants analyzed in the groups to which they were randomized?

Q10. Were outcomes measured in the same way for treatment groups?

Q11. Were outcomes measured in a reliable way?

Q12. Was appropriate statistical analysis used?

Q13. Was the trial design appropriate, and any deviations from the standard RCT design (individual randomization, parallel groups) accounted for in the conduct and analysis of the trial?

# Supplementary Figures


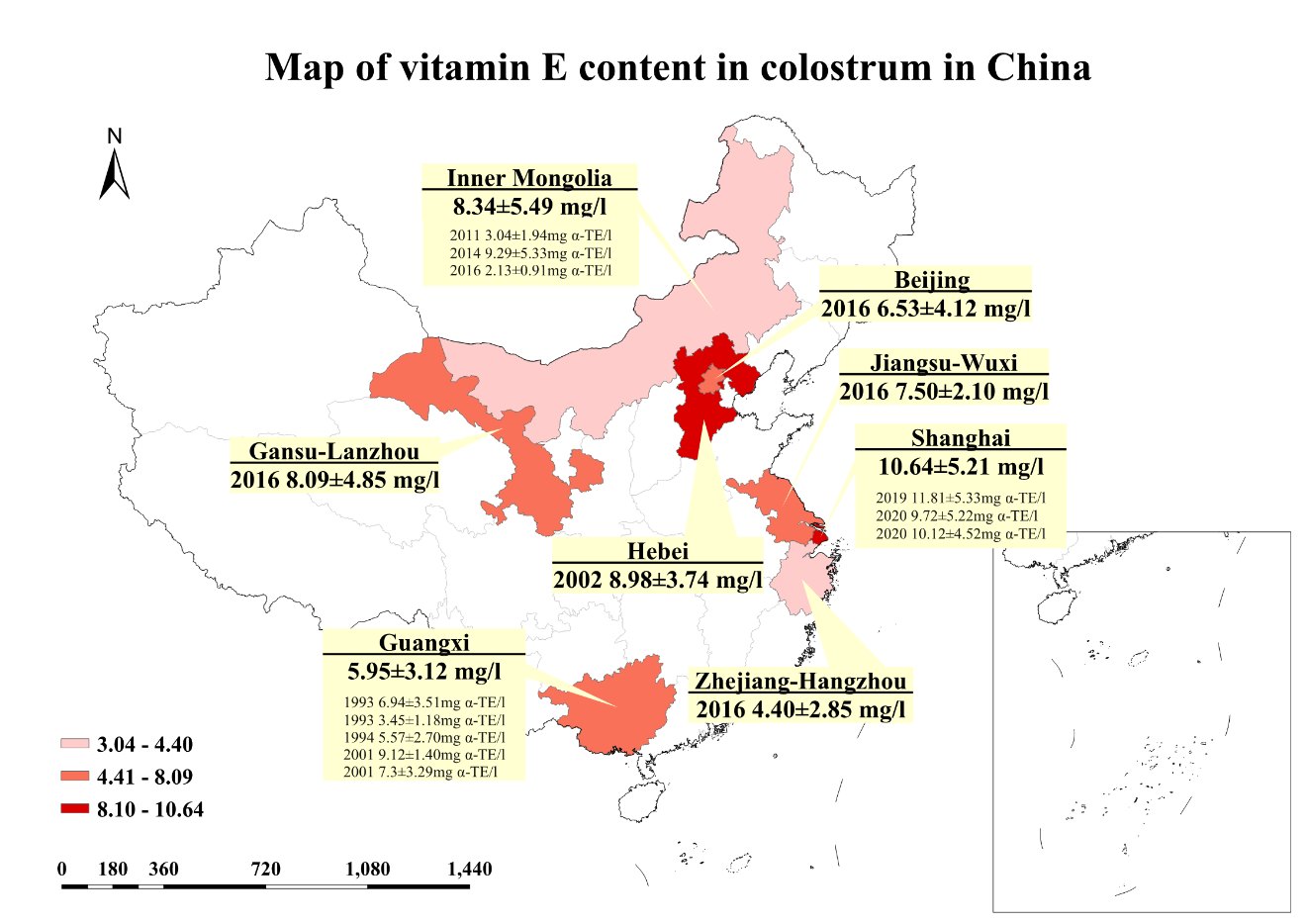


**Supplementary Figure S1.** Map of vitamin E content in colostrum in China
